# Reuse of a Pediatric Liver Graft: A Case Report

**DOI:** 10.1155/2012/350817

**Published:** 2012-11-26

**Authors:** Koray Karabulut, Cengiz Eris, Turgut Piskin, Cuneyt Kayaalp, Sezai Yilmaz

**Affiliations:** Department of Surgery and Liver Transplantation Institute, Inonu University, 44315 Malatya, Turkey

## Abstract

We report the reuse of a liver graft after brain death of the first recipient. The liver donor was an 8-year-old male who died as a result of head injury. The graft was implanted first to a 4-year-old girl for fulminant hepatic failure. Unfortunately she developed progressive coma and brain death on fifth day of transplantation. The graft functions were normal, and reuse of the liver graft was planned. After informed consent, the graft was transplanted to a 31-year-old female recipient who has hepatocellular carcinoma with an underlying cryptogenic liver cirrhosis. The patient was discharged to home on 9th day after an uneventful postoperative period. However, she was readmitted to hospital with an acute abdominal pain 30 days after the operation. Hepatic artery thrombosis was diagnosed, and the attempt to open the artery by interventional radiology was unsuccessful. She died of sepsis and multiorgan failure on 37th posttransplant day.

## 1. Introduction

The shortage of donors in relation to patients on the waiting list requires the consideration of any possible organ donor in order to meet the current demand. Brain death in fulminant hepatic failure is a possible but rare complication after successful orthotopic liver transplantation [1–4]. Previous liver transplant recipients may experience brain death and become organ donors [3, 5]. If liver graft continues to function normally, and there is not any other contraindication, the reuse of liver for another patient may be considered [2, 3]. We report the case of a 4-year-old female donor who had received a liver graft for fulminant hepatic failure due to hepatitis A virus, 5 days before becoming an organ donor herself after brain death.

## 2. Case Report

A 4-year-old girl was admitted to our hospital with fulminant hepatic failure due to hepatitis A virus. She developed progressive coma without any findings of brain death in neurologic examination. Orthotopic liver transplantation was uneventfully performed by using the normal functioning liver of an 8-year-old male cadaveric donor. Cold ischemic time of the graft was 540 minutes. Graft functions were normal after the transplantation, but the patient's neurologic conditions worsened. She was diagnosed as having brain death five days after the transplantation. Reuse of the liver graft was planned. After informed consent, the graft was transplanted to a 31-year-old female patient with hepatocellular carcinoma with an underlying cryptogenic liver cirrhosis. The recipient was 50 kg in weight. Second cold ischemic time was 85 minutes. The patient was discharged to home on postoperative 9th day after an uneventful postoperative period. She was readmitted to hospital with an acute abdominal pain 30 days after the operation. Hepatic artery was occluded on computed tomography scans. The attempt to open the thrombosed hepatic artery by interventional radiology was unsuccessful. She expired due to sepsis and multiorgan failure on 37th posttransplant day.

## 3. Conclusion

There are several ways to enlarge the organ pool for liver transplantation. Living donor liver transplantation, splitting the liver of a deceased donor into two parts, and using grafts from marginal donors are the most popular ways to increase the organ pool. Less used ways are domino transplantation and reutilizing the previously transplanted liver. Brain death in fulminant hepatic failure is a rare complication after successful orthotopic liver transplantation [1, 2]. In this condition, previous recipient may become a potential donor. There are some case reports and small series of cases regarding the successful reuse of liver graft in early and late posttransplant period [1–4, 6, 7]. If the liver functions are normal and there is not any other contraindication, the graft can be reused. 

 To our knowledge, this is the youngest reused liver graft in the literature. Our donor was 4-year old. This may be the reason of late hepatic arterial occlusion. Previously, Rentsch et al. reported a 16-year-old girl (63 kg and 163 cm) as a teenage donor whose liver was successfully used twice [6]. Despite our negative result after reuse of a liver graft, we still believe that previous liver recipients may be considered donor candidates as well. 

## References

[1] Moreno González E, Gómez R, Gonzalez Pinto I (1996). Reuse of liver grafts after early death of the first recipient. *World Journal of Surgery*.

[2] Tantawi B, Cherqui D, Duvoux C, Dhumeaux D, Fagniez PL (1996). Reuse of a liver graft five days after initial transplantation. *Transplantation*.

[3] Castellote J, Lladó L, Xiol X (2006). Successful reuse of liver grafts after death of the first recipient. *Clinical Transplantation*.

[4] Nafidi O, Letourneau R, Willems BE, Lapointe RW (2007). Reuse of liver graft from a brain dead recipient. *Clinical Transplantation*.

[5] Moreno EG, Garcia GI, Gonzalez-Pinto I, Gomez SR, Loinaz SC (1991). Successful reuse of a liver graft. *British Journal of Surgery*.

[6] Rentsch M, Meyer J, Andrassy J (2010). Late reuse of liver allografts from brain-dead graft recipients: the Munich experience and a review of the literature. *Liver Transplantation*.

[7] Tayar C, Karoui M, Laurent A (2006). Successful reuse of liver graft 13 years after initial transplantation. *Transplantation*.

